# The impact of coronavirus disease 2019 on surveillance colonoscopies in South Australia

**DOI:** 10.1002/jgh3.12525

**Published:** 2021-03-09

**Authors:** Molla M Wassie, Madelyn Agaciak, Charles Cock, Peter Bampton, Graeme P Young, Erin L Symonds

**Affiliations:** ^1^ Cancer Research, Flinders Health and Medical Research, Flinders University Bedford Park South Australia Australia; ^2^ Department of Human Nutrition Institute of Public Health, College of Medicine and Public Health, Gondar University Gondar Ethiopia; ^3^ Department of Medicine College of Medicine and Public Health, Flinders University Bedford Park South Australia Australia; ^4^ Department of Gastroenterology and Hepatology, Flinders Medical Centre Bedford Park South Australia Australia; ^5^ Department of Gastroenterology and Hepatology Royal Adelaide Hospital Adelaide South Australia Australia; ^6^ Bowel Health Service, Flinders Medical Centre Bedford Park South Australia Australia

**Keywords:** colorectal neoplasms, coronavirus disease 2019, surveillance

## Abstract

**Background and Aim:**

The coronavirus disease 2019 (COVID‐19) global pandemic has affected elective procedures, including colonoscopy, worldwide. Delayed colorectal cancer surveillance may increase cancer risk. This study aimed to determine the impact of COVID‐19 on the proportion of surveillance colonoscopies booked and completed and the extent to which that surveillance was delayed.

**Methods:**

This was a retrospective analysis of colonoscopy data during the 3 months (April–June 2020) when clinical services were most affected by COVID‐19 in South Australia compared to the same period in 2019. Data on colonoscopies and responses to surveillance recall letters were reviewed to determine the numbers and proportions of colonoscopies that were delayed.

**Results:**

During 2020, the total number of colonoscopies decreased by 51.1% (*n* = 569) compared to 2019 (*n* = 1164). In 2019, 45.5% (*n* = 530) of colonoscopies were completed for surveillance, but this proportion decreased to 32.0% (*n* = 182) during 2020, an overall decrease in the number of surveillance colonoscopies of 65.6%. Of surveillance colonoscopies that were due in 2020, 46.1% (134/291) were delayed >6 months, a significant increase compared to 2019 (19.3%; 59/306, *P* < 0.001). A decrease in response to surveillance recall letters was only observed in patients ≥75 years, with more nonresponders (51.6%) in 2020 compared to that observed in 2019 (25.6%, *P* = 0.03).

**Conclusions:**

Significant delays in surveillance colonoscopies occurred during the COVID‐19 pandemic in South Australia. These effects are likely to be in areas more severely affected by the pandemic. Planning for post‐COVID‐19 colonoscopy capacity is required to avoid cancer progression due to delays in surveillance colonoscopies.

## Graphical abstract

This study investigated the impact of coronavirus disease 2019 (COVID‐19) on surveillance colonoscopies through a retrospective audit of colonoscopy data when clinical services were most affected by COVID‐19 in South Australia (April–June 2020) compared to the same period in 2019. There was a decrease in the number of surveillance colonoscopies during the COVID‐19 pandemic by 66%, resulting in 46% being delayed >6 months. Planning for post‐COVID‐19 colonoscopy capacity is required to avoid cancer progression due to delays in surveillance colonoscopies.

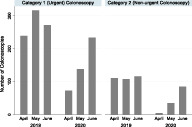

## Introduction

Coronavirus disease 2019 (COVID‐19) is a serious infectious disease that was declared a pandemic by the World Health Organization in March 2020.^1^ The COVID‐19 pandemic has impacted diagnostic procedures, including those used for cancer prevention activities. Many countries ceased cancer screening activities during the height of the pandemic, including the provision of fecal immunochemical tests (FIT) used for colorectal cancer (CRC) screening, as well as limiting the capacity for screening or diagnostic colonoscopy.^2^, ^3^ Such changes have the potential to reduce and delay CRC detection, increasing the proportion of later‐stage cancers diagnosed^4^ and the overall incidence and mortality of CRC.^5^, ^6^

Not only does pandemic‐related restrictions of health services potentially affect average‐risk populations, but it also affects those individuals at an increased risk for CRC, such as those with a personal or family history of neoplasia. For these individuals, regular surveillance colonoscopies, with evidence‐based interval timings, are recommended to reduce the risk for developing CRC.^7^ Endoscopic societies around the world suggested limiting and postponing guideline‐approved elective procedures such as surveillance colonoscopy depending on local pandemic restrictions and available resources.^8^, ^9^ The Gastroenterological Society of Australia suggested prioritizing emergency and urgent colonoscopies, deferring elective colonoscopies, and reviewing other indications on a case‐by‐case basis effective 26th March 2020.^10^ Within South Australia, a location with limited COVID‐19 cases, this still resulted in over 3 months of limitations in endoscopy services due to prioritization.

Limited colonoscopy capacity, as well as patient reluctance to attend hospital, may lead to colonoscopies being delayed. Delays in surveillance colonoscopies might increase the progression of cancer in people at increased risk for CRC.^11^ However, the effects of the COVID‐19 pandemic on the timeliness of surveillance colonoscopies and patient participation rates are currently unknown in Australia.

It is important to understand what impact the pandemic has had on the colonoscopy services and clinical care for patients at higher risk of CRC as this will guide appropriate future strategies. Therefore, this study aimed to determine the impact of COVID‐19 on the number of colonoscopies performed, the magnitude of delay to surveillance colonoscopies, and whether the pandemic altered patient response to a colonoscopy recall letter in South Australia.

## Methods

### *Study design, setting, and participants*

This was a retrospective analysis of surveillance data during the 3 months (April–June 2020) when colonoscopy services were most affected by COVID‐19 in South Australia compared to the same 3 months in 2019 (before COVID‐19). Data for the clinical colonoscopy audit were obtained from two hospitals of the Southern Adelaide Local Health Network (SALHN): Flinders Medical Centre (FMC), an acute care hospital, and Noarlunga Health Service (NHS), a community‐based hospital. These two centers share the public colonoscopy workload in Southern Adelaide, including surveillance colonoscopies on patients under the Southern Cooperative Program for the Prevention of Colorectal Cancer (SCOOP).^12^

Data on when surveillance colonoscopies were recommended, if and when such colonoscopies occurred, and responses to letters to arrange the colonoscopy surveillance recall were obtained from the SCOOP program clinical records.^12^ SCOOP is a guidance‐based, senior nurse‐led hospital surveillance program that coordinates surveillance colonoscopy for people who are at elevated risk for CRC. The surveillance colonoscopy interval is recommended based on the national guidelines,^7^ and patient colonoscopy details, along with the due date (i.e. recall date) for the next surveillance colonoscopy, are maintained within a centralized clinical database. Patients (<75 years) are sent a colonoscopy recall letter up to 3 months before the procedure is due. This letter requests that the patient contact a SCOOP nurse for a health interview via telephone. After the interview, the patient is booked for his or her colonoscopy procedure. The process differs for patients ≥75 years as current Australian guidelines suggest that once individuals reach this age, ongoing surveillance colonoscopy may no longer be appropriate. Therefore, for these patients, a letter is sent to the individual and his or her general practitioner to consider whether further surveillance colonoscopy is appropriate. After discussing with their general practitioner, patients may choose to withdraw from further participation by notifying their withdrawal.

All colonoscopies performed within the public hospitals of the SALHN in the audit periods were included. Whether colonoscopies due in the audit period in public hospitals of SALHN (FMC and NHS) occurred or not was determined to examine potential delays in colonoscopies. If they did not occur when due, the length of the delay was determined. Surveillance colonoscopies performed earlier than their due date because of a positive FIT result or symptoms were excluded from consideration.

The study was registered as a quality improvement project with the Southern Adelaide Clinical Human Research Ethics Committee (quality register ID 2116).

### *Assessment of outcomes*

An assessment of all colonoscopies completed during April, May, and June of 2019 and 2020 was undertaken, with a review of the number of colonoscopies and the indications for each procedure. The data were extracted from each hospital's colonoscopy database. The indications were categorized into the following groups: surveillance, positive screening FIT, symptoms, abnormal abdominal radiology, inflammatory bowel disease (IBD), and other indications. For patients with multiple indications of colonoscopy, we considered every indication as a stand‐alone indication. The indications for the colonoscopies were further divided into category 1 (urgent) or category 2 “ready for care” based on an urgency triage model practiced by the audited hospitals. Category 1 procedures aimed to be completed within 30 days and included indications of positive screening FIT, alarming symptoms (iron deficiency anemia, rectal bleeding, active diarrhea, and change in bowel habit), abnormal abdominal radiology, and suspected active IBD (assessment of disease activity or treatment response). Patients who were scheduled for surveillance colonoscopies but presented with alarming symptoms were treated as category 1 based on urgency. Category 2 procedures aimed to be completed within 90 days and included mainly indications of surveillance (including postcurative resection of CRC, personal history of adenoma, and family history of CRC or IBD for >8 years [IBD CRC surveillance]) but could also include some nonalarming symptoms (chronic/persistent diarrhea and change in bowel habit). For individuals with multiple indications, the overall category was considered the most urgent.

A review of the data related to surveillance colonoscopies was carried out using data extracted from the SCOOP clinical database to assess whether surveillance colonoscopies were completed close to their recommended due date and to assess patient responses to the recall letter. Surveillance colonoscopies that were due during the audited months were assessed for the proportions that were delayed in 2019 and 2020. Procedures were defined as delayed if the colonoscopy was performed more than 3 months after the due date, based on national guideline,^13^ and more than 6 months after the due date, based on literature.^11^

To determine whether the pandemic altered the patient response to a recall letter for surveillance colonoscopy, the proportion (who were <75 years) that responded to the letter, and the time taken to respond were compared before and during COVID‐19. The responses of the patients ≥75 years after they had been sent a letter to consider the need for further surveillance colonoscopy (as described above) were assessed to determine the proportion requesting ongoing colonoscopy, and those who did not respond to the letter, during 2019 and 2020.

### *Statistical analysis*

The data were analyzed using State 16.0. The number of colonoscopies before and during the COVID‐19 pandemic was compared by the site (FMC *vs* NHS) and month (April *vs* May *vs* June). The chi‐square (*χ* ^2^) and Fisher's exact (when *n* < 10) tests were used to compare frequencies and percentages between groups. The time taken to respond to the colonoscopy recall letter was expressed as median and interquartile range (IQR), with results compared with Mann–Whitney test. A *P* value less than 0.05 was considered statistically significant.

## Results

### *Number of colonoscopies before and during the pandemic*

**Figure 1:**
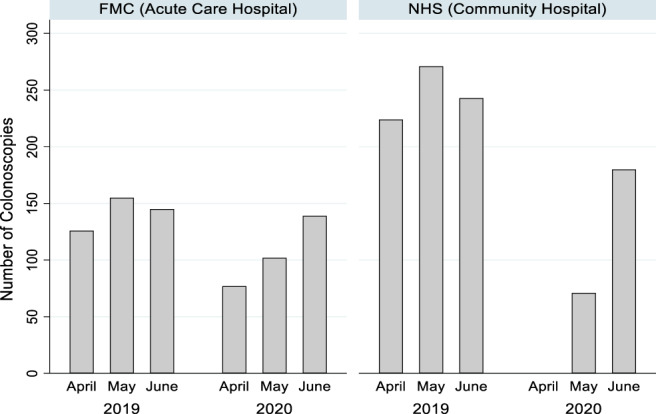
Number of colonoscopies performed in April, May, and June of 2019 and 2020 in Flinders Medical Centre and Noarlunga Health Service.

During the audited period in 2020, the total number of colonoscopies completed decreased by 51.1% (*n* = 569) compared to the same months in 2019 (*n* = 1164), with the greatest decrease of 88% observed in April 2020. As shown in Figure 1, there was a complete shutdown of colonoscopies in NHS in April of 2020, but colonoscopies resumed at reduced numbers in May 2020. FMC continued functioning at reduced numbers throughout the pandemic.

### *Proportion of colonoscopy indications before and during the pandemic*

The recorded indications for the completed colonoscopies are shown in Table 1. Overall, in 2019, 45.5% (*n* = 530/1164) of the colonoscopies had an indication suggestive of surveillance. This proportion of colonoscopies completed for surveillance indications decreased to 32.0% (*n* = 182/569) in 2020, resulting in an overall decrease of 65.7% in the number of completed colonoscopies with surveillance as an indication (*P* < 0.001).

**Table 1:** Summary of colonoscopy procedures before and during coronavirus disease 2019 periods

	April			May			June					
Indicators	2019 (*n*, %)	2020 (*n*, %)	*P* ^†^	2019 (*n*, %)	2020 (*n*, %)	*P* ^†^	2019 (*n*, %)	2020 (*n*, %)	*P* ^†^	2019 (*n*, %)	2020 (*n*, %)	*P* ^†^
N	350	77		426	173		388	319		1164	569	
Surveillance	147 (42.0)	7 (9.1)	<0.001^‡^	193 (45.3)	53 (30.6)	0.001	190 (49.0)	122 (38.2)	0.004	530(45.5)	182(32.0)	<0.001
Positive FIT	73 (20.9)	18 (23.4)	0.63	85 (19.9)	54 (31.2)	0.003	75 (19.3)	68 (21.3)	0.43	233 (20.0)	140 (24.6)	0.03
Symptomatic	110 (31.4)	38 (49.3)	0.003	127 (29.8)	53 (30.6)	0.84	112 (28.9)	113 (35.4)	0.06	349 (30.0)	204 (35.9)	0.01
IBD	23 (6.6)	5 (6.5)	0.98^‡^	27 (6.3)	11 (6.4)	0.99	22 (5.7)	17 (5.3)	0.84	72 (6.2)	33 (5.8)	0.75
Abnormal abdominal radiology	8 (2.3)	13 (16.9)	<0.001	9 (2.1)	8 (4.6)	0.09^‡^	15 (3.9)	11 (3.4)	0.77	32 (2.8)	32 (5.6)	0.003
Others	33 (9.4)	9 (11.7)	0.55^‡^	39 (9.1)	22 (12.7)	0.19	37 (9.5)	17 (5.3)	0.04	109 (9.4)	48 (8.4)	0.53

^†^ Pearson chi‐square test unless otherwise specified.

^‡^ Fisher's exact test.

Some colonoscopies had more than one indication recorded.

FIT, fecal immunochemical test; IBD, inflammatory bowel disease.

The changes between the months can be seen in Table 1. Compared to April 2019, the proportion of surveillance colonoscopies decreased, while colonoscopies performed for symptoms or due to abnormal abdominal radiology increased in the month of April in the COVID‐19 period (April 2020) (*P* < 0.05). The proportion of colonoscopies performed for a positive FIT, IBD assessments, or symptoms did not vary before and during the pandemic (*P* > 0.05) except a slight proportional increase in FIT positive tests in the month of May during the pandemic (*P* = 0.003).

### *Urgent and nonurgent colonoscopies before and during the pandemic*

**Figure 2:**
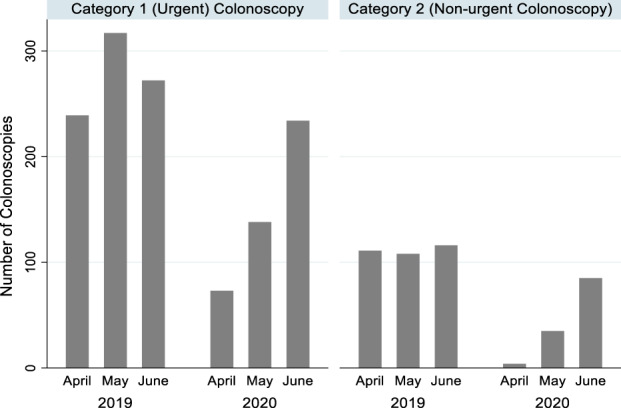
Urgent and nonurgent colonoscopies before and during the coronavirus disease 2019 pandemic.

The proportion of urgent colonoscopies increased from 71.2% (828/1163) in 2019 to 78.2% (445/569) in 2020, accompanied by a significant decrease in the number of nonurgent colonoscopies completed from 335 to 124 (63.0% reduction, *P* = 0.002). Even though the total number of completed nonurgent colonoscopies significantly decreased during the pandemic, this increased from 4 in April to 35 in May and had reached 73% (*n* = 85) of the average 2019 capacity by June (Fig. 2).

### *Proportion of delayed surveillance colonoscopies before and during the pandemic*

There were 597 surveillance colonoscopies due during the audited period, including 22.6% that had been recalled after a 3‐year surveillance (*n* = 135) and 36.5% that had been recalled after a 5‐year interval (*n* = 218). As the due dates are previously determined up to 5 years based on previous colonoscopy pathology outcomes, the total numbers of colonoscopies due were similar in 2019 (*n* = 306) and 2020 (*n* = 291). Of these, the number of surveillance colonoscopies that were due but had not been completed with 3 months of this due date increased from 52.9% (162/306) in 2019 to 68.0% (198/291) in 2020 (*P* < 0.001), and the number of surveillance colonoscopies that had not been completed within 6 months of the due date increased from 19.3% (59/306) in 2019 to 46.1% (134/291) in 2020 (*P* < 0.001). During the COVID‐19 period, the percentage of surveillance colonoscopies delayed by 3 months was higher in the month of April when compared with May and June (Table 2). Many of the delayed procedures were for those patients with 3‐ and 5‐year surveillance recall intervals during and before the COVID‐19 pandemic (Table S1, Supporting information).

**Table 2:** Comparison of delayed surveillance colonoscopy in the before and during coronavirus disease 2019 (COVID‐19) periods

	3‐month delay			6‐month delay		
Months	Before COVID‐19 (*n*, %)	During COVID‐19 (*n*, %)	*P* value^†^	Before‐COVID‐19 (*n*, %)	During COVID‐19 (*n*, %)	*P* value^†^
April	43 (55.0)	59 (86.8)	<0.001	13 (16.7)	35 (51.5)	<0.001
May	52 (42.3)	71 (62.8)	0.002	21 (17.1)	48 (42.5)	<0.001
June	67 (63.8)	68 (61.8)	0.763	25 (23.8)	51 (46.4)	0.001

^**†**^ Pearson chi‐square test.

### *Patient response to recall letter for surveillance colonoscopy*

The median time taken to respond to the recall colonoscopy letter was comparable in 2019 (21 days, IQR 12–48 days) and 2020 (18 days, IQR 11–41 days) (*P* = 0.23). Moreover, there was no significance difference in the number of nonrespondents in the COVID‐19 era (34/162, 21.0%) compared with the same months in 2019 (18/102, 17.6%) (*P* = 0.44).

### *Response to recall letter in the* *SCOOP* *patients ≥75 years*

**Figure 3:**
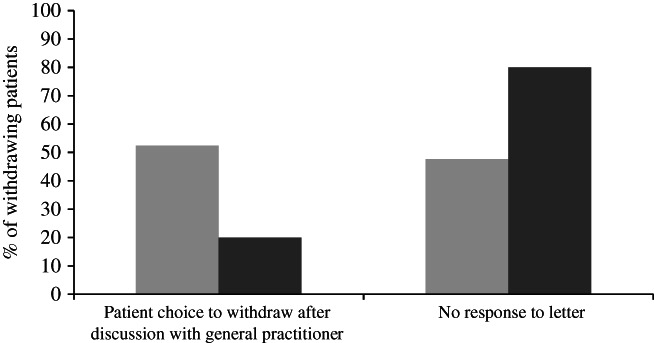
Reasons for withdrawing from the Southern Cooperative Program for the Prevention of Colorectal Cancer program and having no further surveillance colonoscopies after age 75 years. 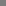, 2019; 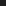, 2020.

For the patients ≥75 years sent a letter to consider another colonoscopy, in 2020, there were significantly more nonresponders (16/31, 51.6%) compared to that observed in 2019 (10/39, 25.6%, *P* = 0.03); however, for responders there was no difference in the proportion requesting booking (62.1% in 2019 and 73.3% in 2020, *P* = 0.46). Fewer patients engaged in a discussion with their general practitioners regarding ongoing colonoscopy and were thus withdrawn from the SCOOP program during the COVID‐19 period (Fig. 3).

## Discussion

This study is the first to examine the impact of the COVID‐19 pandemic on colonoscopy procedures in South Australia, a region and country affected by the pandemic in a limited way. This study showed significant reductions in numbers, and consequent delays, in surveillance colonoscopies in South Australia. However, no significant changes were observed in the majority of patient responses to surveillance colonoscopy recall letters other than reduced responses by older participants.

The impact of the pandemic on surveillance colonoscopies in South Australia is much lower than in Europe^2^, ^4^ and the United States^14^, ^15^ during a similar time period. Over the 3‐month audited period in South Australia, there was a decrease in the number of completed colonoscopies by 51%. Rutter et al. reported an overall 89.7% reduction in colonoscopies in the United Kingdom between March and May 2020.^4^ Cancer referrals and diagnoses also decreased in the Netherlands.^16^ In Italy, CRC screening was completely suspended in 46.7% of gastroenterology units.^2^ The main impact of these reductions has been observed on the nonurgent colonoscopies, with the audit in the United Kingdom showing a reduction in the number of these procedures by 95%.^4^ Within Australia, a majority of the nonurgent colonoscopies is completed for surveillance purposes, with our study showing that there was a 63% reduction in the number of completed nonurgent surveillance colonoscopies compared to 2019.

We investigated whether the reduction in surveillance colonoscopies was just a reflection of the overall reduction in colonoscopy capacity or whether it was also contributed to by patient reluctance to book a procedure. Previous studies show that decreases in hospital presentations are due to patient reluctance to visit a health‐care center for fear of infection.^17^, ^18^, ^19^ While we observed that this may be the case in patients ≥75 years, who had an increase in their nonresponse rate to a letter from the SCOOP program, the majority of patients (and who are <75 years) did not significantly change their booking behavior. This could be because, as part of the SCOOP program, the visits to the hospital associated with the booking are limited; the patient does not need to attend a precolonoscopy appointment, and interviews are conducted via telephone. In addition, the patient may be less fearful of booking a colonoscopy for 3 months in the future, thinking that the spread of COVID‐19 may be reduced by then.

The majority of the colonoscopies performed during the pandemic was for symptoms and positive FIT results, both regarded as urgent indications. Surveillance procedures were disproportionately affected by reduced service delivery. Although colonoscopy is an aerosol‐generating procedure, and attending health‐care facilities for the colonoscopy procedure increases the potential for COVID‐19 transmission,^20^ urgent colonoscopies were still performed in SALHN hospitals during the pandemic in order to not compromise patient care and with the knowledge of a very limited pandemic in South Australia. Some surveillance was still performed, for instance, following a recent past cancer diagnosis and particularly after colonoscopy removal of advanced neoplasia. A model such as SCOOP stratifies increased risk through a database of past findings, with the most recent findings of either cancer or advanced neoplasia^12^, ^21^ being most relevant to the next procedure due. This model can be adapted to countries affected to a greater extent by the pandemic to ensure that those at greatest risk are serviced within a limited capacity.

Our study showed a higher number of delayed surveillance colonoscopies during the pandemic. In the pre‐COVID‐19 period, the usual proportions of colonoscopies completed for surveillance were about half of all colonoscopy procedures. In the SCOOP program, as more people are identified at elevated risk (either from a family or personal history of CRC and current adenoma), more people are going to need ongoing surveillance colonoscopy. Therefore, even without colonoscopy restrictions, strategies are needed to reduce the number of surveillance colonoscopies and prioritize urgent colonoscopies. Tinmouth et al.^22^ suggested redirecting the lower‐risk surveillance colonoscopies to FIT as a strategy to reduce colonoscopy backlog. FIT between colonoscopy has been found to be effective in detecting advanced colorectal lesions between colonoscopies.^23^ Within the audited period of the current study, just over one‐third of patients scheduled for surveillance colonoscopy were considered to be at the lowest risk as they had been given a 5‐year interval. These patients could be redirected to FIT, helping to reduce the burden on the limited resources. A detailed and individualized risk assessment strategy to determine which patients can safely undergo less frequent colonoscopies is required.

Delays in surveillance colonoscopy were observed at both short and long surveillance intervals, although the majority of the patients with delayed colonoscopy had a 3‐ or 5‐year interval. Previous studies have shown that delays in screening, diagnostic, and surveillance colonoscopies increase the risk for CRC progression and mortality.^24^, ^25^, ^26^, ^27^ The literature shows that delays of 6 months and above are associated with later‐stage CRC when screening with FIT.^11^ Most of the patients in the study period had a less than 6‐month delay and may not pose a problem for disease progression. However, long‐term follow‐up is required to observe the colonoscopy outcomes of these patients. Surveillance colonoscopies are prevention strategies with proven effectiveness in preventing CRC progression and improving cancer survival in elevated risk patients.^28^ Thus, planning for post‐COVID‐19 surveillance colonoscopy triage and capacity is required to avoid cancer progression in elevated‐risk patients due to delays in surveillance colonoscopies.

The study has the following limitations. First, the study focused on numbers and indications of colonoscopies rather than pathology outcomes and ways to catch up. The audit period was not long enough, and case numbers are insufficient to assess any increase in the stage of neoplasia at diagnosis in those patients with delayed surveillance colonoscopies. Second, the audit was conducted in hospitals with an organized surveillance program, which limits the generalizability of the data on the patient responses to being recalled for surveillance colonoscopy.

## Conclusions

Significant reductions and delays in surveillance colonoscopies were seen during the COVID‐19 pandemic in South Australia despite a very limited pandemic in this geographic location. This occurred due to a reduction in the total number of nonurgent procedures rather than patient reluctance to undergo their procedure. These effects are likely to be much larger in areas significantly affected by the pandemic. Thus, planning for post‐COVID‐19 colonoscopy triage and capacity is required to avoid cancer progression in elevated‐risk patients due to delays in surveillance colonoscopies.

## Author contributions

Molla M Wassie, Charles Cock, Graeme P Young, and Erin L Symonds designed the study. Molla M Wassie and Erin L Symonds performed the analysis and drafted the manuscript. Erin L Symonds oversaw the study conduct and critically reviewed the manuscript. Madelyn Agaciak contributed to the data analysis. Charles Cock, Graeme P Young, Madelyn Agaciak, and Peter Bampton contributed to interpretation of results and reviewed the manuscript. All authors approved the final manuscript.

## Supporting information


**Table S1** Characteristics of surveillance colonoscopy delay by surveillance interval.
