# Supplementary material for: The impact of coronavirus disease 2019 on surveillance colonoscopies in South Australia
Source: JGH Open. 2021 Mar 9;5(4):486–92. doi: 10.1002/jgh3.12525 (PMC8035475; doi:10.1002/jgh3.12525)
Supplement: Supplementary file 1 — Table S1 Characteristics of surveillance colonoscopy delay by surveillance interval. [file JGH3-5-486-s001.docx]

**Supplemental Table 1:** characteristics of surveillance colonoscopy delay by surveillance interval

| Delay | | Surveillance interval | | |  |
| --- | --- | --- | --- | --- | --- |
|  | | <12 months (n, %) | 36 months (n, %) | 60 months (n, %) | P^†^ |
| 3 months | 2019 | 20/109 (18.4) | 53/77 (68.8) | 89/120 (74.2) | <0.001 |
|  | 2020 | 51/135 (37.8) | 51/58 (87.9) | 96/98 (98.0) | 0.03 |
| 6 months | 2019 | 13/109 (11.9) | 15/77 (19.5) | 31/120 (25.8) | <0.001 |
|  | 2020 | 34/135 (25.2) | 38/58 (65.5) | 62/98 (63.3) | <0.001 |

^†^Pearson chi-square test
